# Systematic review and meta-analysis for the proximal junctional kyphosis in adolescent idiopathic scoliosis

**DOI:** 10.3389/fped.2024.1387841

**Published:** 2024-08-14

**Authors:** Jian Zhao, Chen Huang, Yifei Liu, Da Liu, Dongfa Liao

**Affiliations:** ^1^ Department of Orthopedics, The General Hospital of Western Theater Command, Chengdu, Sichuan, China; ^2^ Department of Pain Medicine, The General Hospital of Western Theater Command, Chengdu, Sichuan, China

**Keywords:** proximal junctional kyphosis, AIS, adolescent idiopathic scoliosis, meta-analysis, complication

## Abstract

**Objective:**

The risk factors of PJK (proximal junctional kyphosis) related to AIS (adolescent idiopathic scoliosis) are inconsistent due to heterogeneity in study design, diagnostic criteria, and population. Therefore, the meta-analysis was conducted to investigate the factors affecting PJK after posterior spinal fusion for AIS patients.

**Methods:**

We implemented a systematic search to obtain potential literature relevant to PJK in AIS surgery. Then, a meta-analysis was performed to assess the incidence of PJK and its risk factors.

**Results:**

We retrieved 542 articles, and 24 articles were included. The PJK incidence was 17.67%. The use of hooks at UIV (upper instrumented vertebrae) (*p* = 0.001) could prevent PJK. Before surgery, the larger TK (thoracic kyphosis) (*p* < 0.001), GTK (global thoracic kyphosis) (*p* < 0.001), and LL (lumbar lordosis) (*p* < 0.001) were presented in the PJK group. Immediately post-operatively, in the PJK group, the following parameters were higher: TK (*p* = 0.001), GTK (*p* < 0.001), LL (*p* = 0.04), PJA (proximal junctional angle) (*p* < 0.001), and PJA-RCA (rod contouring angle) (*p* = 0.001). At the final follow-up, the following parameters were higher in the PJK group: TK (*p* < 0.001), GTK (*p* < 0.001), LL (*P* < 0.001), and PJA (*P* < 0.001). Sub-group analysis detected that before surgery, the following parameters were larger in the PJK group: TK (*p* < 0.001), LL (*p* = 0.005), and PJA (*p* = 0.03) in Lenke type 5 AIS patients. Immediately post-operatively, in the PJK group, the following parameters were higher: TK (*p* < 0.001), LL (*p* = 0.005), and PJA (*p* < 0.001). At the final follow-up, the following parameters were higher in the PJK group: TK (*p* < 0.001), LL (*p* < 0.001), and PJA (*p* < 0.001).

**Conclusion:**

The individuals with larger preoperative TK were more susceptible to PJK, and PJA was mainly influenced by the adjacent segments rather than the whole sagittal alignment. Using hooks or claws at UIV should prevent PJK.

## Introduction

Adolescent idiopathic scoliosis (AIS) is the most common spinal deformity, and females aged 10 to 18 are more susceptible to AIS (1). It is widely recognized that AIS can lead to appearance abnormalities (2), psychological disorders (3), cardiopulmonary dysfunction (4), and so on. Its treatment is a comprehensive process, which requires consideration of Cobb angle, curve shape, growth potential, and other factors. Generally, clinical observation is recommended for patients with a Cobb angle of less than 20 degrees, and for patients with a Cobb angle between 20 and 40 degrees, brace treatment is recommended (5). Even through, it is reported that bracing is also effective for large curves of higher than 40 degrees in this patient population that forcefully reject the surgical treatment and insist on to use a brace (6), correction surgery is commonly recommended (7, 8). With the application of pedicle screws and osteotomy technology, we can reconstruct the spine alignment in coronal, sagittal and transverse planes (9). However, the corresponding complications cannot be ignored such as proximal junctional kyphosis (PJK), rod breakage, and pseudoarthrosis (10–12).

PJK is a common complication after long-segment spinal internal fixation. In 1994, Lowe et al. (13) firstly used the concept of junctional kyphosis to describe the phenomenon of PJK and distal junction kyphosis (DJK) after posterior spinal fusion (PSF) in Scheuermann's disease. However, it did not provide diagnostic criteria for PJK. Referencing the physiological curvature of the spine, Lee et al. (14) proposed the PJK diagnostic criteria in 1999 as follows: the kyphotic angle between the upper endplate of T2 vertebrae and the lower endplate of upper instrumented vertebrae (UIV) was more than 5 degrees higher than the physiological kyphosis. Yang et al. (15) defined PJK as the local kyphosis angle increased by 10° compared with that immediately after surgery. The measurement method is the angle between the UIV upper endplate and the UIV + 2 lower endplate (15). At present, the widely used measurement and diagnostic criteria were put forward by Glattes et al. (16) in 2005: the kyphotic angle between the lower endplate of UIV and the upper endplate of UIV + 2 was greater than 10°, which is more than 10° higher than that before surgery. The recently reported PJK diagnostic criteria include that the angle between UIV and UIV + 1 is greater than 15 degrees (17), and the angle between UIV and UIV + 2 is greater than or equal to 20 degrees (18). Initially, the studies reported that PJK was only an imaging change that had little relationship with clinical outcomes (13). Nowadays, it is accepted that PJK can cause back pain, severe deformity, and nerve compression symptoms (19). In particular, the concept of proximal junction failure (PJF), combined with the high surgical revision rate (20), has got more attention to PJK and PJF.

Given the different definitions of PJK and the heterogeneity of samples, the incidence varied from 3.22% to 46% (21–28) in AIS patients. The Lenke type 5 curve AIS was more susceptible to PJK (29). Correspondingly, a series of literature reported the influencing factors of PJK. The influencing factors of PJK can be roughly divided into three categories: (1) patient factors, such as bone maturity, gender, curve shape, preoperative Cobb angle; (2) factors related to surgical procedures, such as rod material, whether the posterior ligament is resected, whether the UIV uses the lamina hook, fusion segments; (3) postoperative spinal alignment, such as whether there is flat back deformity and whether there is a mismatch of PI-LL. However, the results reported in different studies are inconsistent. For example, Kim et al. (30) reported that the hybrid construct (proximal hooks) was a risk factor for PJK, while Ogura et al. (31) reported that using hooks at UIV might prevent PJK. Several studies reported that the larger preoperative thoracic kyphosis (TK) was related to PJK (27, 32–34), while Kim et al. and Chen et al. reported that the preoperative TK was not associated with PJK. In terms of preoperative lumbar lordosis(LL), Albay et al. (34) and Ferrero et al. (28) detected a larger LL in the PJK group, while other studies reported no difference in LL (22, 23, 35).

Overall, there are many reports about PJK after AIS, and the risk factors reported in different studies are inconsistent due to heterogeneity in study design, diagnostic criteria, and population. Therefore, the meta-analysis was conducted to investigate the rate of PJK and its risk factors in AIS populations after PSF.

## Materials and methods

### Literature retrieval and screening

To ensure the quality of the research, every step of the research strictly followed the standard Preferred Reporting Items for Systematic Reviews and Meta-Analyses (36). To obtain potential literature relevant to PJK in AIS surgery, we implemented a systematic search based on the following abstract databases including PubMed, Web of Science, and Embase December 12th, 2023. The following were the search terms: (proximal junctional kyphosis) AND (scoliosis) AND (adolescent OR pediatric).

### Inclusion and exclusion criteria

Inclusion criteria: (1) case-control, retrospective, or cohort study designs based on AIS patients; (2) posterior spinal fusion was performed for each case; (3) the study mainly reported the incidence and risk factors of PJK;(4) the diagnostic criteria of PJK was that the kyphotic angle between the lower endplate of UIV and the upper endplate of UIV + 2 was greater than 10°, which is more than 10° higher than that before surgery (16); (5) the literature was written in English. Exclusion criteria: (1) the study focused on other types of scoliosis; (2) anterior spinal fusion was performed; (3) the diagnostic criteria of PJK did not meet that the kyphotic angle between the lower endplate of UIV and the upper endplate of UIV + 2 was greater than 10°, which is more than 10° higher than that before surgery (16); (4) the literature was not written in English. According to the inclusion criteria, the two authors independently judge the inclusion or exclusion of the literature. If there is a dispute about the inclusion of a certain literature, the research group will decide whether to include it in this meta-analysis through discussion and voting. The Newcastle-Ottawa quality assessment scale (NOQA) was used to assess the quality for each paper.

### Data collection

The following items were extracted from papers meeting the inclusion criterion: (1) the author's name, publication year, study design, follow-up period, age, and gender distribution; (3) radiographic parameters: thoracic kyphosis (TK), global thoracic kyphosis (GTK) lumbar lordosis (LL), pelvic tilt (PT), pelvic incidence (PI), pelvic incidence minus lumbar lordosis (PI-LL), sagittal vertical axis (SVA), proximal junctional angle (PJA), RCA(rod contouring angle); (4) Surgery details: the fusion segments, screws at UIV, UIV hooks at UIV.

### Publication bias

The Begg's test was conducted and the funnel plot was employed to evaluate the publication bias.

### Sensitivity analysis

Sensitivity analyses were performed by omitting each included paper in sequence.

### Statistical analysis

The statistics were pooled using the Review Manager Version 5.3. For the enumeration data, the odds ratio (OR) with the 95% confidence interval (95%CI) was calculated, while the weight mean difference (WMD) with the 95%CI was employed to assess the difference of measurement data between groups. That *P < 0.05* meant the statistical significance between PJK and Non-PJK groups. That *I^2^* ≥ 50% meant that the random effect model should be used, while the fixed effect model would be used when *I^2^* < 50%.

## Results

### Paper selection and characteristics

**Figure 1:**
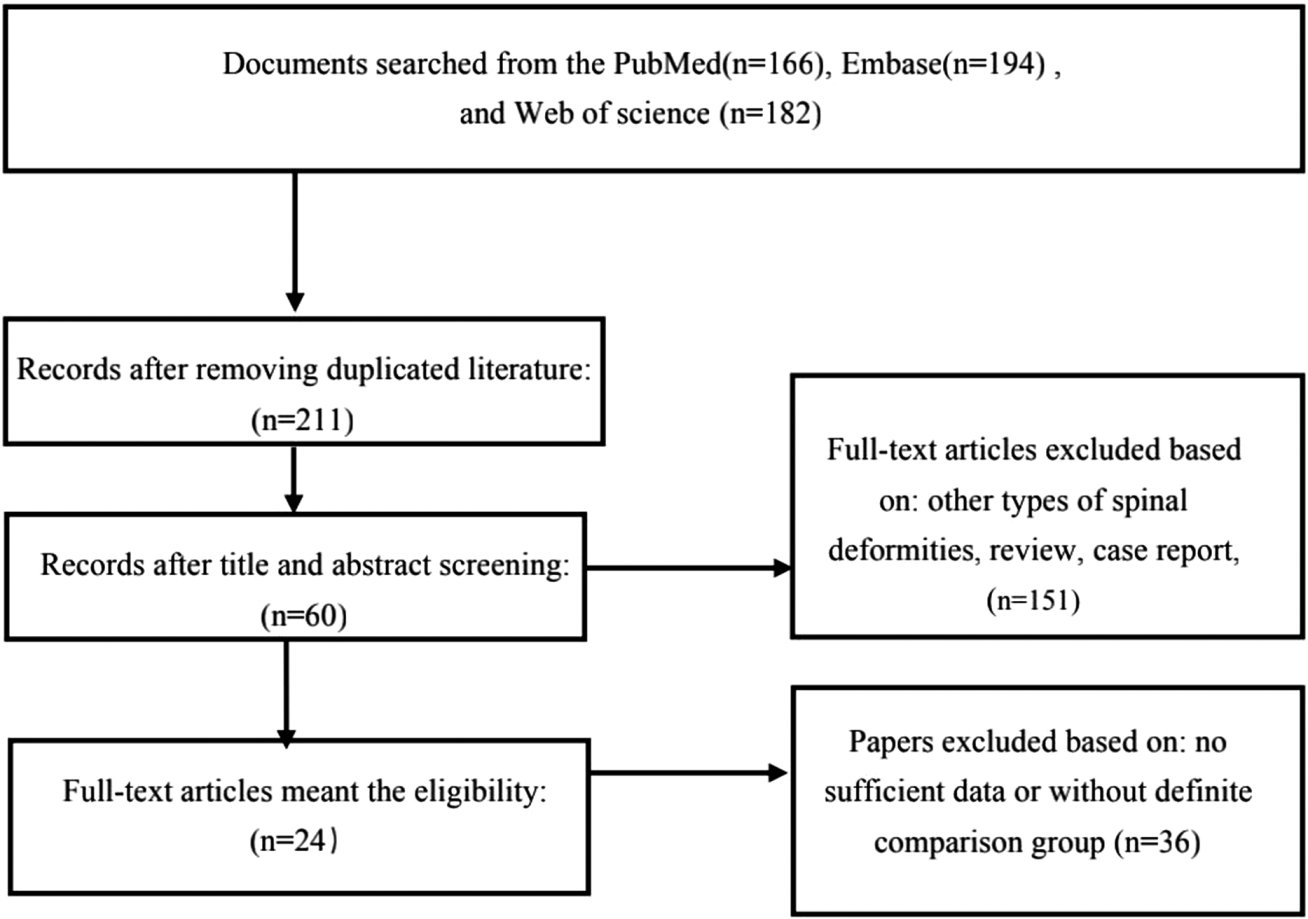
The flow chart showed the details process of paper selection.

Through systematic retrieval of the databases, 542 articles were retrieved. Among them, 24 articles met the inclusion criteria (21–29, 31–35, 37–46). Four studies groups were based on Lenke 1/2 AIS patients (26–28, 44), and 8 studies were based on Lenke 5 AIS patients (21–25, 34, 35, 46), while the remaining studies did not clearly distinguish different types of AIS when investigate the incidence of PJK and its influencing factors (29, 31–33, 37–43, 45) (Table 1). Figure 1 demonstrates the details of the literature screening procedures. A total of 4,063 AIS cases were included in this meta-analysis, and the incidence of PJK was 17.67% for the whole population (PJK = 718, non-PJK = 3,885). However, the corresponding rate of PJK inclined to 23.88% in Lenke type 5 AIS patients (PJK = 166, non-PJK = 529).

**Table 1:** The basic information of the included research literatures.

First author	Publication year	Sample		Age (years)	Follow-up time (years)	Lenke type (1/2/3/4/5/6)
		PJK	Non-PJK			
Wang et al.	2023	33	173	PJK: 14.9 ± 2.3 Non-PJK: 14.4 ± 2.3	PJK: 2.8 ± 1.4 Non-PJK: 2.7 ± 1.3	PJK:20/13/0/0/0/0 Non-PJK: 101/72/0/0/0/0
Erkilinc et al.	2023	30	307	14.2 ± 1.9	3.14 ± 1.01	215/5/29/0/61/27
Coury et al.	2023	17	177	14.9 ± 5	2∼5	0/0/0/0/149/28
Luhmann et al.	2022	25	75	14.6 ± 2.1	3.9 ± 1.6	PJK: 9/2/5/2/4/3 Non-PJK: 39/12/10/4/5/5
Boeckenfoerde et al.	2022	30	139	PJK:16.9 ± 8.66 Non-PJK:16.1 ± 4.36	14.7 ± 6.25	Unavailable
Hu et al.	2022	23	75	PJK: 15.3 ± 2.6 Non-PJK: 15.7 ± 2.6	3.12 (2-3)	0/0/0/0/98/0
Albay et al.	2022	41	74	14.6 ± 2.9	4.82 ± 2.29	0/0/0/0/115/0
Ogura et al.	2021	15	330	PJK: 14.5 + 2.1 Non-PJK: 14.5 + 2.2	PJK: 2.0 + 0.9 Non-PJK: 2.2 + 1.3	PJK: 5/2/3/1/0/4 Non-PJK: 108/92/44/31/27/28
Kim et al.	2021	7	62	PJK: 13.9 ± 0.9 Non-PJK: 14.2 ± 2.2	PJK: 9.4 ± 2.8 Non-PJK: 8.3 ± 3.5	PJK: 2/0/0/0/5/0 Non-PJK: 41/21/1/4/6/8
Clément et al.	2021	102	468	PJK: 15.6 Non-PJK: 15.1	PJK: 9.4 ± 2.8 Non-PJK: 8.3 ± 3.5	PJK: 59/24/8/1/0/10 Non-PJK: 292/125/19/9/0/23
Langlais et al.	2021	2	58	16 ± 2	a minimum of 2 years’ follow-up	40/0/20/0/0/0
Zhou et al.	2021	13	57	15.3 ± 2.1	3,01 ± 1.29	0/0/0/0/70/0
Wang et al.	2021	12	40	14.0 ± 1.8	2.9 ± 0.9	0/0/0/0/52/0
Chen et al.	2021	15	20	PJK: 15.7 ± 1.9 Non-PJK: 15.7 ± 2.1	3.58（2 = 7.67）	0/0/0/0/35/0
Clément et al.	2020	7	77	PJK: 14.7 ± 1.8 Non-PJK:15.0 ± 2.6	2.9 (2∼8.2)	37/23/3//7/11/3
Wang et al.	2020	20	64	PJK: 14.60 ± 1.43 Non-PJK: 14.55 ± 1.28	2	Lenke 1/2
Peng et al.	2020	10	34	18.27 ± 3.61	3.15 ± 2.67	0/0/0/0/44/0
Alzakri et al.	2019	13	72	15.6 ± 1.99	a minimum of 2 years’ follow-up	
Ferrero et al.	2018	57	308	15 ± 2.6	2.5 ± 0.4	296/69/0/0/0/0
Zhao et al.	2018	35	52	13.85 ± 1.49	4.67 ± 1.17	0/0/0/0/87/0
Lonner et al.	2017	60	791	14.4	a minimum of 2 years’ follow-up	394/182/58/23/106/88
Ghailane et al.	2017	5	45	14.8 (11.6–22.4)	1.5 (0.83–2.17)	25/2/17/2/0/4
Wang et al.	2010	35	88	PJK: 15.0 (13.0, 16.0) Non-PJK: 15.0 (13.0, 16.0)	3.5	Unavailable
Kim et al.	2007	111	299	PJK: 14.5 ± 1.83 Non-PJK: 14.8 ± 2.03	2 years’ follow-up	195/76/51/13//31/44

### Meta-analysis results for the whole population

There was no difference in age at surgery (*p* = 0.47), gender distribution (*p* = 0.1), BMI (*p* = 0.52), Risser sign (*p* = 0.37), or follow-up time (*p* = 0.89) between groups. However, the pooled results showed more fusion segments (WMD = 0.49, 95% = 0.29∼0.68, *p < 0.001*) in PJK groups. Compared with the use of screws at UIV, the use of hooks (OR = 0.48, 95% = 0.31∼0.47, *p* = 0.001) could prevent PJK (Table 2).

**Table 2:** The pooled results between the between the PJK and Non-PJK group for the whole population.

Variables	Test of difference			Model	Heterogeneity	
	WMD/OR	95%CI	*P*-value		*P*-value	Chi^2^(%)
Preoperative age	−0.12	−0.33∼0.09	0.47	F	0.79	0
Gender (female vs. male)	0.83	0.64∼1.09	0.19	F	0.48	0
BMI	0.18	−0.37∼0.73	0.52	F	0.52	0
Risser sign	−0.10	−0.32∼0.12	0.37	F	0.47	0
Follow-up time	0.32	−4.36∼5.00	0.89	R	0.07	48
Fusion segments	0.49	0.29∼0.68	<0.001	F	0.29	18
UIV hooks versus screw	0.48	0.31∼0.74	0.001	F	0.09	47

F, fix effect model; R, random effect model.

**Figure 2:**
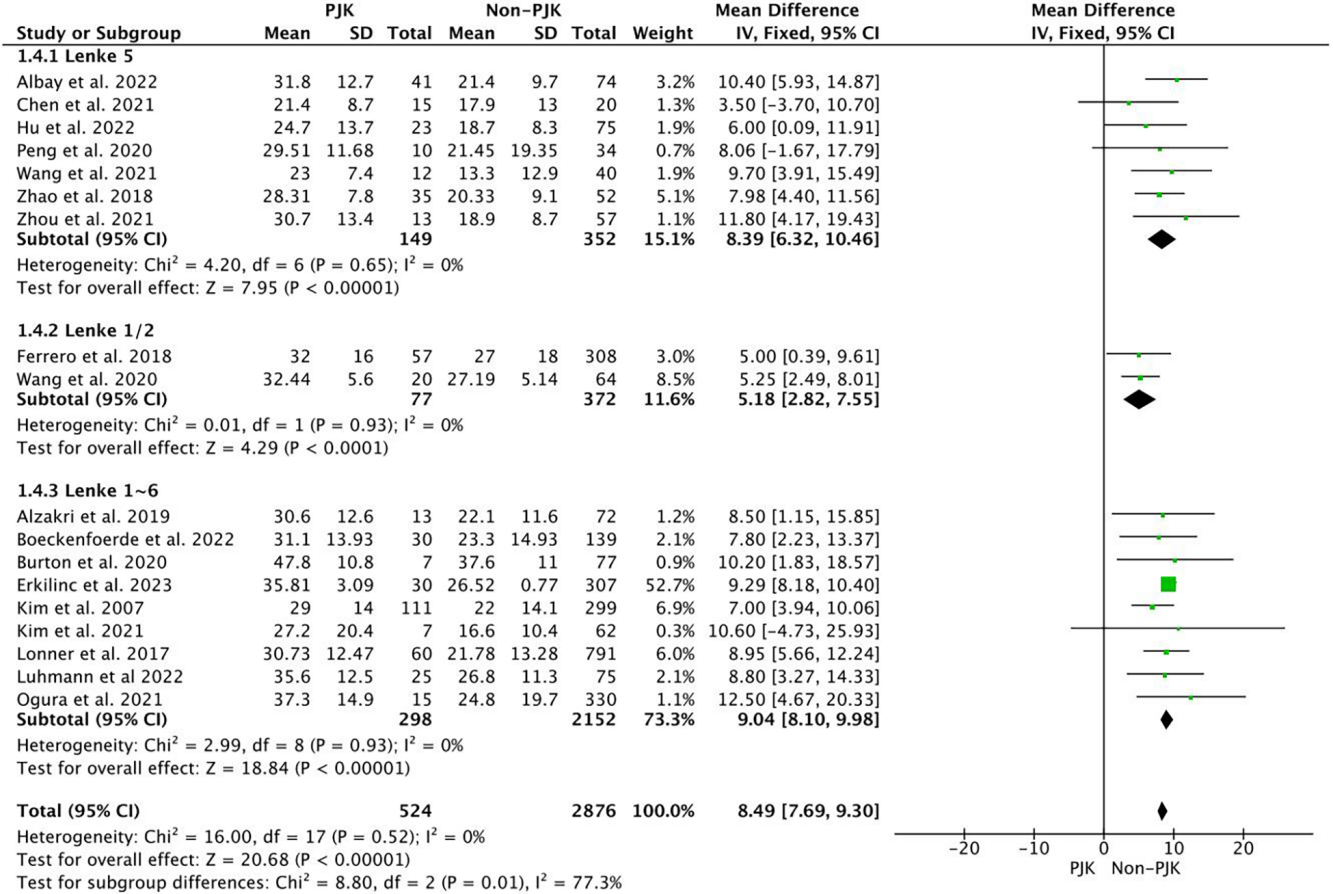
The pooled result showed that the PJK had a larger TK before surgery.

Before surgery, the larger TK (WMD = 8.49, 95%CI = 7.69∼.30, *p < 0.001*), and GTK (WMD = 10.1, 95%CI = 5.55∼14.66, *p < 0.001*) were observed in PJK group, and a lager LL (WMD = 4.11, 95%CI = 3.40∼4.82, *p < 0.001*) was also presented in PJK group (Figure 2). For the pelvic parameters, a smaller PT (WMD = −1.02, 95%CI = −1.85∼−0.18, *p = 0.02*) was observed in the PJK group. However, there was no difference in PI (*p* = 0.16), SS (*p* = 0.38), PI-LL (*p* = 0.14), SVA (*p* = 0.65), RCA (*p* = 0.39), and PJA (*p* = 0.94).

**Figure 3:**
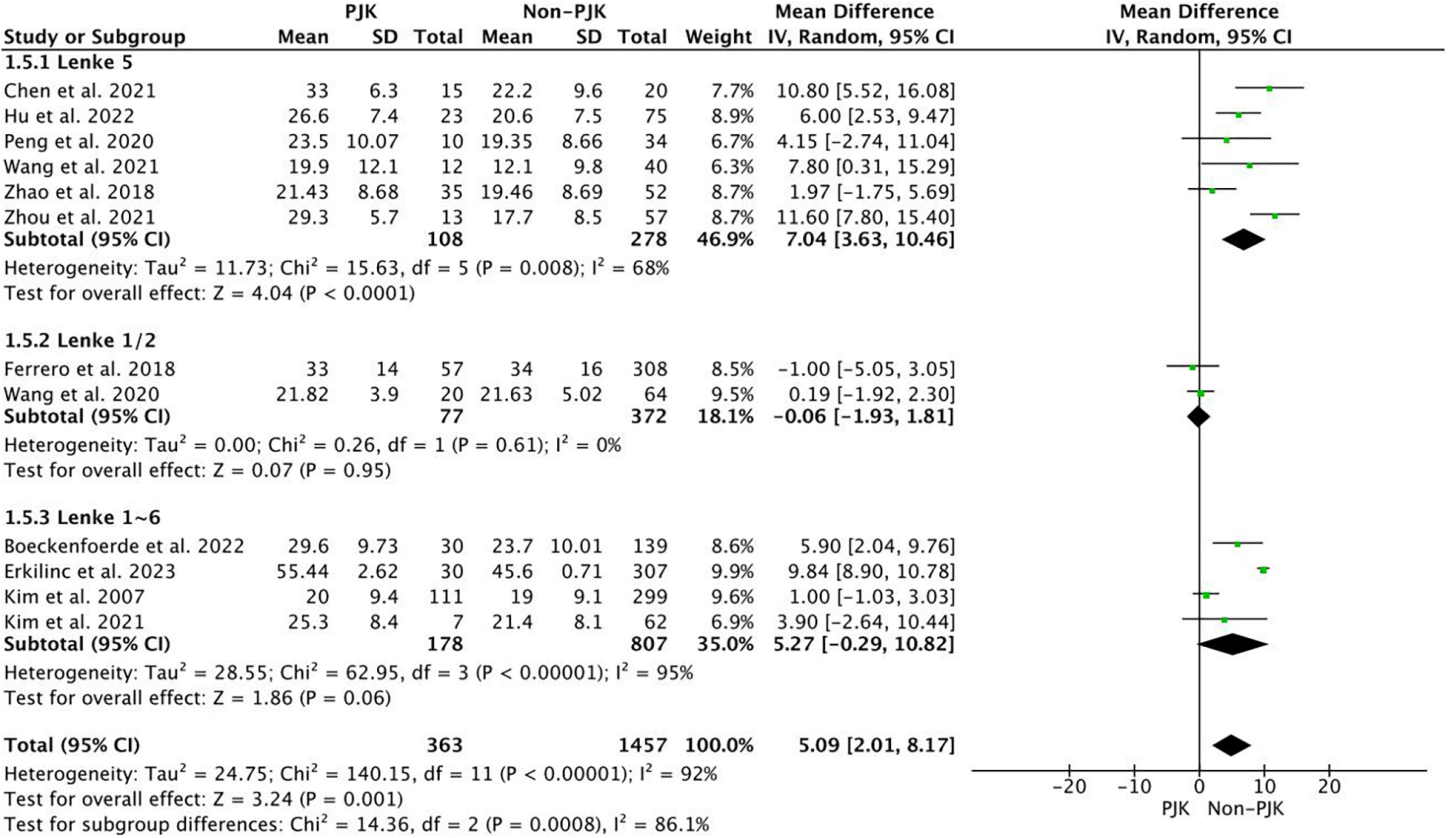
The pooled result showed that the PJK had larger TK before and immediately after surgery.

Immediately postoperatively, the pooled results demonstrated higher values in the PJK group for the following parameters: TK (WMD = 5.09, 95%CI = 2.01∼8.17, *p* *= 0.001*), GTK (WMD = 10.07, 95%CI = 7.05∼13.09, *p < 0.001*), LL (WMD = 3.53, 95% CI = 0.21∼6.85, *p = 0.04*), PJA (WMD = 5.00, 95%CI = 4.03∼5.94, *p < 0.001*), and PJA-RCA (WMD = 3.89, 95%CI = 1.53∼6.25, *p* *= 0.001*) (Figure 3). However, a smaller PI-LL (WMD = −7.52, 95%CI = −14.79∼−0.25, *p = 0.04*) was observed in the PJK group, and no difference was demonstrated in PI (*p = 0.34*), PT (*p = 0.15*), SS (*p = 0.80*) and SVA (*p = 0.06*).

At the final follow-up, the following parameters were higher in the PJK group: TK (WMD = 5.13, 95%CI = 4.09∼6.16, *p < 0.001*), GTK (WMD = 12.30, 95%CI = 8.16∼15.99, *p < 0.001*), LL (WMD = 3.33, 95%CI = 2.09∼4.56, *P < 0.001*), and PJA (WMD = 13.20, 95%CI = 11.06∼15.34, *P < 0.001*). However, a smaller PI-LL (WMD = −11.65, 95%CI = −17.48∼−5.81, *p < 0.001*) was observed in the PJK group, and no difference was demonstrated in PI (*p = *0.19), PT (*p = 0.05*), SS (*p = 0.77*) and SVA (*p = 0.97*) (Table 3).

**Table 3:** Comparison of the radiographic parameters between the PJK and Non-PJK group for the whole population.

Variables	Test of difference			Model	Heterogeneity	
	WMD/OR	95%CI	*P*-value		*P*-value	Chi^2^
Preoperative						
TK	8.49	7.69∼9.30	<0.001	F	0.52	0
GTK	10.1	5.55∼14.66	<0.001	F	0.68	0
LL	4.11	3.40∼4.82	<0.001	F	0.85	0
PI	−1.51	−3.88∼0.85	0.21	R	<0.001	80
PT	−1.02	−1.85∼−0.18	0.02	F	0.09	37
SS	−0.77	−2.47∼0.94	0.38	R	0.04	50
PI-LL	−5.08	−11.85∼1.69	0.14	R	<0.001	87
SVA	1.56	−5.14∼8.26	0.65	R	<0.001	65
PJA	−0.03	−0.87∼0.81	0.94	R	0.01	58
RCA	0.78	−1.01∼2.57	0.39	R	<0.001	74
Immediate post-operation						
TK	5.09	2.01∼8.17	0.001	R	<0.001	92
GTK	10.07	7.05∼13.09	<0.001	F	0.29	12
LL	3.53	0.21∼6.85	0.04	R	<0.001	88
PI	−2.06	−6.31∼2.19	0.34	R	<0.001	90
PT	−1.93	−4.55∼0.68	0.15	R	<0.001	74
SS	−0.21	−1.87∼1.45	0.80	F	0.54	0
PI-LL	−7.52	−14.79 ∼−0.25	0.04	R	<0.001	87
SVA	3.85	−0.09∼7.79	0.06	F	0.17	31
PJA	5.00	4.03∼5.94	<0.001	R	0.03	53
PJA-RCA	3.89	1.53∼6.25	0.001	R	0.10	64
Final follow-up						
TK	5.13	4.09∼6.16	<0.001	R	<0.001	92
GTK	12.30	8.16∼15.99	<0.001	F	0.75	0
LL	3.33	2.0∼4.56	<0.001	F	0.46	0
PI	−2.90	−7.24∼1.43	0.19	R	<0.001	86
PT	−2.26	−4.55∼0.03	0.05	R	0.002	68
SS	−0.18	−1.38∼1.03	0.77	F	0.19	31
PI-LL	−11.65	−17.48∼−5.81	<0.001	R	<0.001	81
SVA	0.12	−0.63∼6.62	0.97	R	0.01	59
PJA	13.20	11.06∼15.34	<0.001	R	<0.001	89

F, fix effect model; R, random effect model.

### Sub-group analysis in lenke type 5 AIS patients

Before surgery, the following parameters were larger in the PJK group: TK (WMD = 8.39, 95%CI = 6.32∼10.46, *p < 0.001*), LL (WMD = 5.14, 95%CI = 2.88∼7.39, *p = 0.005*), and PJA (WMD = 0.79, 95%=0.07∼1.50, *p = 0.03*). For the pelvic parameters, a smaller PT (WMD = −2.10, 95%CI = −3.47∼−0.73, *p = 0.003*) was observed in the PJK group. However, there was no difference in PI (*p* = 0.05), SS (*p* = 0.43), PI-LL (*p* = 0.18), and SVA (*p* = 0.58).

Immediately post-operatively, the pooled results demonstrated higher values in the PJK group for the following parameters: TK (WMD = 7.04, 95%CI = 3.63∼10.46, *p* *= 0.02*), LL (WMD = 3.06, 95%CI = 0.90–5.22∼−0.90, *p = 0.005*), and PJA (WMD = 5.54, 95%CI = 3.57∼7.52, *p < 0.001*). However, a smaller PI-LL (WMD = −9.76, 95%CI = −17.90 ∼−1.63, *p = 0.02*) was observed in the PJK group, and no difference was demonstrated in PI (*p = 0.33*), PT(*p = 0.09*), SS (*p = 0.80*) and SVA (*p = 0.58*).

At final follow-up, the following parameters were higher in the PJK group: TK (WMD = 9.51, 95%CI = 5.03∼13.99, *p < 0.001*), LL (WMD = 4.75, 95%CI = 2.57∼6.93, *P < 0.001*), and PJA (WMD = 11.47, 95%CI = 8.21∼14.74, *P < 0.001*). However, a smaller PI-LL (WMD = −13.23, 95%CI = −19.70∼−6.75, *p < 0.001*) and a smaller PT (WMD = −3.70, 95%CI = −6.75∼−0.66, *p = 0.02*) were observed in PJK group, and no difference was demonstrated in PI (*p = *0.12), SS (*p = 0.90*) and SVA (*p = 0.56*) (Table 4).

**Table 4:** Comparison of the radiographic parameters between the PJK and Non-PJK group in lenke type 5 AIS patients.

Variables	Test of difference			Model	Heterogeneity	
	WMD/OR	95%CI	*P*-value		*P*-value	Chi^2^
Preoperative						
TK	8.39	6.32∼10.46	<0.001	F	0.65	0
LL	5.14	2.88∼7.39	0.005	F	0.37	6
PI	−6.34	−12.78∼0.10	0.05	F	<0.001	79
PT	−2.10	−3.47∼−0.73	0.003	F	0.11	42
SS	−0.60	−2.11∼0.90	0.43	F	0.13	40
PI-LL	−6.14	−15.11∼2.83	0.18	R	<0.001	84
SVA	1.71	−4.37∼7.78	0.58	F	0.15	38
PJA	0.79	0.07∼1.50	0.03	F	0.38	2
Immediate post-operation						
TK	7.04	3.63∼10.46	<0.001	R	<0.001	68
LL	3.06	0.90∼5.22	0.005	F	0.37	6
PI	−9.39	−28.39∼9.62	0.33	R	<0.001	94
PT	−3.86	−8.34∼0.63	0.09	R	<0.001	80
SS	−0.21	−1.87∼1.45	0.80	F	0.54	0
PI-LL	−9.76	−17.90 ∼−1.63	0.02	R	<0.001	82
SVA	1.71	−4.37∼7.78	0.58	F	0.15	38
PJA	5.54	3.57∼7.52	<0.001	R	0.005	73
Final follow-up						
TK	9.51	5.03∼13.99	<0.001	R	<0.001	77
LL	4.75	2.57∼6.93	<0.001	F	0.36	8
PT	−3.70	−6.75∼−0.66	0.02	R	0.03	63
PI	−7.07	−16.04∼1.90	0.12	R	<0.001	88
SS	0.11	−1.58∼1.81	0.90	F	0.59	0
PI-LL	−13.23	−19.70∼−6.75	<0.001	R	0.004	78
SVA	−2.85	−12.32∼6.62	0.56	F	0.24	28
PJA	11.47	8.21∼14.74	<0.001	R	0.002	80

F, fix effect model; R, random effect model.

### Clinical outcome

There was no significant difference in the preoperative (*p* = 0.18) and postoperative SRS-22 scores (*p* = 0.46) between the PJK group and the non-PJK group.

### Publication bias

**Figure 4:**
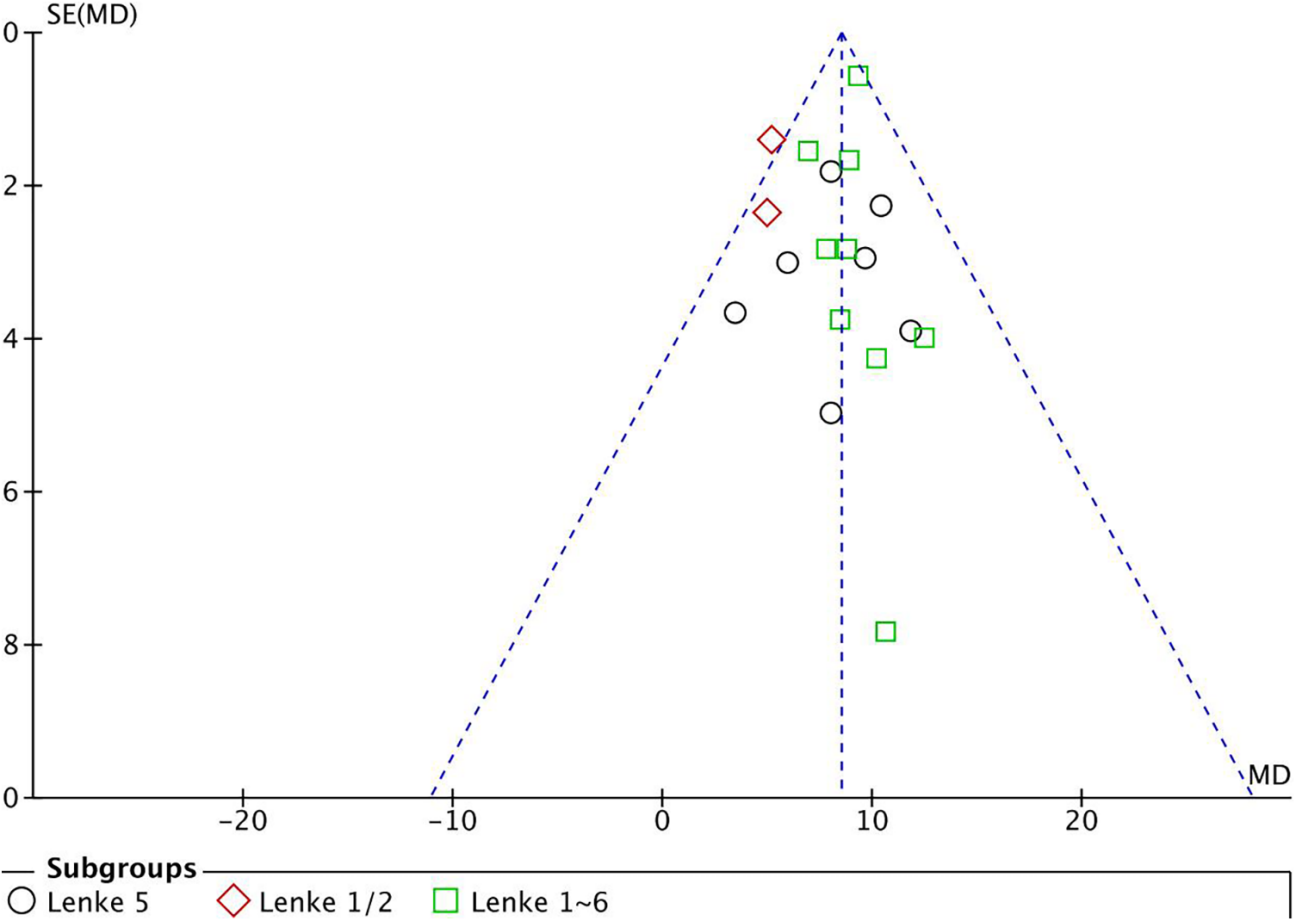
The funnel plot was symmetrical, which indicated no significant publication bias in this meta-analysis.

Based on the preoperative TK Begg's test was performed, and a symmetrical funnel graph was obtained. So, there was no significant publication bias in this meta-analysis (Figure 4).

### Sensitivity analysis

No single paper resulted in huge fluctuations in the pooled results.

## Discussion

### The rate of PJK in AIS individuals after PSF

The incidence of PJK reported in previous studies fluctuates greatly. Based on the diagnostic criteria of PJK by Glattes (16), the highest PJK rate varied from 3.33% to 42.86% (23, 26). While the majority of papers reported that the rate of PJK was 15∼30% (32, 33, 40–42). The current meta-analysis detected an incidence of PJK to be 16.50% for the whole population, while the corresponding figure inclined to 23.88% in Lenke type 5 AIS patients. Previously, several studies also reported that Lenke type 5 AIS patients were more susceptible to PJK after PSF with the rates varying from 18.57% to 42.86% (21–25, 34, 35). Therefore, the PJK phenomenon in AIS should be paid enough attention.

### Radiographic factors associated with PJK in AIS individuals after PSF

Previous studies have investigated various influencing factors that might be related to PJK in AIS patients. Among all the factors, the most reported ones are radiographic factors, and the current research mainly investigated the influence of sagittal spinopelvic parameters on PJK. It is commonly accepted that the UIV always locates in the thoracic segments, which means that the TK is always composed of PJA. Before surgery, Boeckenfoerde et al. (38) reported that the PJK group had significantly larger T4–T12 kyphosis (31.1° ± 13.93° vs. 23.3° ± 14.93°, *p* = 0.016). Kim et al. (30) reported that the preoperative TK > 40° was one of the risk factors for PJK. Currently, the pooled results also demonstrated the larger TK (WMD = 8.49, 95%CI = 7.69∼.30, *p < 0.001*), and larger GTK (WMD = 10.1, 95%CI = 5.55∼14.66, *p* < 0.001) in PJK group. Correspondingly, other studies also detected a more kyphotic thoracic spine in the PJK group before surgery (32, 33, 38). The subgroup analysis also detected a larger TK in the PJK group in Lenke type 5 AIS patients, which was also consistent with several previous reports (21, 22, 25, 34). Generally, the greater the thoracic kyphosis, the greater the lumbar lordosis. Ferrero et al. (28) and Clément et al. (39) reported that the preoperative LL was significantly greater in the PJK group. However, several reports did not detect differences in preoperative LL between groups (22–24, 32, 34, 38). The pooled result detected a larger preoperative LL (WMD = 3.53, 95%CI = 0.21∼6.85, *p = 0.*04) in the PJK group, and a similar result was also presented in subgroup analysis LL (*p* *= 0.005*). So, it is necessary to take appropriate measures to prevent PJK for patients with large TK before surgery, especially for those with TK greater than 40 degrees (30).

The pooled results detected that both TK and LL in the PJK group were greater in the non-PJK group immediately after operation and at the last follow-up. After PSF, the patient's thoracic kyphosis and lumbar lordosis are not completely developed and shaped. It was commonly accepted that the formation of physiological curvature of the thoracic kyphosis and lumbar lordosis involved the whole spinal segments. Correction surgery fixed several segments which were relatively stable after surgery. This was bound to interfere with the formation of thoracic or lumbar curvature. So, the proximal unfused segments may increase kyphosis in compensation for the development of thoracic kyphosis, which may be the potential mechanism of the increasing PJA after surgery. However, it was reported that the increase in PJA might mainly compensate for the SVA increase in adult spinal deformity patients, while the decompensation led to PJK (47). Therefore, there may be some differences in the main mechanism of PJK in different age groups after surgery.

In terms of pelvic parameters, only a smaller preoperative PT was detected in the PJK group, which was consistent with the reports by Wang et al. (22) and Chen et al. (23). At the last follow-up, subgroup analysis also detected a smaller PT in the PJK group. On the contrary, one of the main influencing factors of PJK is the larger preoperative PT in adult spinal deformity, and the follow-up PT was larger in the PJK group. This again showed that there were differences in the main influencing factors and evolution process of PJK between AIS and adult spinal deformity patients. PJK-related theory, which is applicable to explain adult degenerative spinal deformity, cannot be used to clarify the PJK phenomenon in AIS patients. In adult spinal deformity, correction surgery mainly reconstructed the SVA, LL, and PT, while these parameters kept deteriorating with aging. The fixed segments failed to compensate for the sagittal spinal misalignment, and the increased PJA could compensate for the trunk forward (47). However, in AIS patients the proper sagittal balance is always maintained both before and after surgery and the PJA increase might be a manifestation of thoracic kyphosis. Furthermore, the larger PJA-RCA was detected in the PJK group, which also reflected that the PJA was mainly influenced by the adjacent segments rather than the whole sagittal alignment (27).

### Surgery factors associated with PJK in AIS individuals after PSF

In order to prevent PJK after surgery for adult spinal deformity, surgeons tried many surgical strategies, such as vertebral augmentation, ligament strengthening, semi-rigid structure at the proximal construct, and transverse process hook at UIV (47). However, there are not so many preventive measures in the AIS population. The most reported PJK prevention measure after PSF in AIS is to use lamina hook or transverse claws for UIV, and the reported results are inconsistent (28, 31, 40). The pooled results showed that the use of hooks for UIV (OR = 0.48, 95% = 0.31∼0.47, *p* = 0.001) could prevent PJK. In addition to proximal implants, other surgical-related PJK influencing factors have also been reported, such as disruption of junctional ligaments (25), exposure of adjacent joint (48), and thoracoplasty (42).

### Clinical outcome in AIS individuals after PSF

One of the main reasons why the phenomenon of PJK is getting more and more attention is that PJK can lead to poor clinical outcomes, which has been widely confirmed in adult degenerative spine deformity patients (49). However, for AIS, the researchers did not detect that PJK would have a significant impact on its clinical outcome (25, 35). These pooled results also did not find any difference in the preoperative and postoperative SRS-22 scores between the PJK group and the non-PJK group. The reason for this result may be that the current follow-up time is too short to show the effect of osteoporosis and muscle degeneration on the accelerated deterioration of PJK. We cannot be blindly optimistic that PJK patients after AIS still have no clinical symptoms after aging.

### Limitations

Even though the current meta-analysis retrieved papers on PJK after PSF in AIS patients, the following shortcomings should be taken into consideration. Firstly, the sample sizes were small for some papers included in this meta, which might lead to heterogeneity. Secondly, the current meta-analysis could not perform the subgroup analysis based on different curve types. Finally, only retrospective studies were included, and further prospective studies were needed to detect the primary risk factor of PJK in AIS.

## Conclusion

PJK was a common complication after PSF in AIS. The individuals with larger preoperative TK were more susceptible to PJK, and PJA was mainly influenced by the adjacent segments rather than the whole sagittal alignment. Using hooks or claws at UIV should be one measure to prevent PJK in AIS individuals.
